# Geographical Accessibility of Community Health Assist Scheme General Practitioners for the Elderly Population in Singapore: A Case Study on the Elderly Living in Housing Development Board Flats

**DOI:** 10.3390/ijerph15091988

**Published:** 2018-09-12

**Authors:** Ong Ming Lee Deborah, Marcus Yu Lung Chiu, Kai Cao

**Affiliations:** 1 Department of Geography, National University of Singapore, Singapore, Singapore; 2 Department of Social and Behavioural Sciences, City University of Hong Kong, Hong Kong, China

**Keywords:** geographical accessibility, Healthcare services, GIS, E2SFCA, CHAS, Singapore

## Abstract

Accessible primary healthcare is important to national healthcare in general and for older persons in particular, in societies where the population is ageing rapidly, as in Singapore. However, although much policy and research efforts have been put into this area, we hardly find any spatial perspective to assess the accessibility of these primary healthcare services. This paper analyzes the geographical accessibility of one major healthcare service in Singapore, namely, General Practitioners (GPs) services under the Community Health Assist Scheme (CHAS) for older persons. A Python script was developed to filter the website data of the Housing Development Board (HDB) of Singapore. The data derived was comprehensively analyzed by an Enhanced 2-Step Floating Catchment Area (E2SFCA) method based on a Gaussian distance-decay function and the GIS technique. This enabled the identification of areas with relatively weak geographical accessibility of CHAS-GPs. The findings are discussed along with suggestions for health practitioners, service planners and policy makers. Despite its initial nature, this study has demonstrated the value of innovative approaches in data collection and processing for the elderly-related studies, and contributed to the field of healthcare services optimization and possibly to other human services.

## 1. Introduction

It is widely recognized that primary healthcare services are critical to national healthcare and play a vital role in support of elderly population [1,2,3]. Accessibility to healthcare services is variable across space and time, so it is affected by where health professionals are located (supply) and where people reside (demand) [4]. Besides, the value of healthcare professionals is dependent on the geographical accessibility of healthcare services [5], which depends on the impact of spatial gap between the supply and the demand sides of healthcare resource. In this regard, Geographical Information Systems (GIS) as well as spatial analysis technologies are particularly useful in addressing these kind of issues [6].

Rapid ageing is a key demographic challenge impacting Singapore. The ratio of seniors aged over 65 years, changed from 3.2% in 1970, to 13.0% in 2017, and will reach 24.1% in 2030 and 28.2% (over 900,000) in 2050 [7]. In addition to the challenge arising from the dramatic fall in Old-Age Support Ratio, demand for social and healthcare services is also rapidly increasing [8,9]. When home-based support and family caregiving are preferred to institutional care, policies of various types have been developed to enable seniors to age in place. In the case of Singapore, government seeks to maintain good health among older persons through health and exercise campaigns, building senior-friendly housing and towns, creating senior-friendly communities, and making quality and affordable healthcare services available.

Availability and accessibility of healthcare services is one of the major objectives of Singapore’s national health plans and strategies in Singapore. Singapore’s government is planning to add around 11,000 community hospitals and nursing home beds by 2020. In addition, the government is planning to expand home and community care with nursing homes as back up. One may assume that these additional resources will be accessible to meet the increasing need of the ageing population. However, this assumption may not be valid as revealed in other studies. Cheng [10] found that the distribution of the elderly population and residential care resources is geographically uneven across the districts in Beijing, so the supply of resources does not match with the needs. Similar studies have also been carried out in Hong Kong [11] and metro Atlanta [12]. It should therefore be beneficial to conduct a study on primary healthcare services in Singapore and examine how far the new provisions are meeting the changing spatial and temporal demand [13].

One common approach for assessing physical accessibility is to consider potential spatial accessibility. This approach has been used frequently while analyzing primary healthcare facilities in relation to the population of a catchment area on the basis of a variable of distance [14] that takes the availability as a supply-demand ratio within a region. However, it has been criticized for ignoring spatial variations within administrative boundaries as it assumes that people within the region do not seek healthcare services outside the boundaries [4,15]. Being an extension of accessibility-based approach, the regional accessibility approach considers the contribution and interactions of supply and demand between different regions [16,17]. By contrast, the gravity model integrates both the regional availability and regional accessibility as a unified measure of healthcare services accessibility. This measure has been widely employed in a variety of related studies [18,19].

In spite of the above mentioned advances in spatial analysis, most of the existing studies of the elderly in Singapore have focused on health and socio-economic correlates or determinants [20,21,22,23,24,25,26]. There have been some related studies, e.g., Wong, Heng [27], who used GIS to identify service gaps and suggest optimal sites for future polyclinics in Singapore. Koh, Leow [28] have also studied the mobility of the elderly in densely populated neighbourhoods in Singapore through a survey. Their study reflects more the perception of elderly subjects rather than objective effort to investigate the geographical accessibility of healthcare services for the elderly in Singapore.

In view of this dearth of spatial analysis for Singapore, this paper aims to assess the state of geographical accessibility of primary healthcare services for the elderly living in HDB flats in Singapore. This study is methodologically apt to the city-state of Singapore that is geographically confined with about 3 million resident populations.

## 2. Methodological Considerations

The early versions of a floating catchment area method have been used to measure geographical accessibility to calculate job accessibility. This is done by using the concept of a kernel density model estimation where the events within the kernel were used to represent the density at the center [29,30]. This has been considered a superior method for measuring geographical accessibility because it takes into consideration the cross-boundary demand and the distance decay effect [5,31].

Another popular method is the two-step floating catchment area (2SFCA) method, which is a gravity based model that takes into account both the accessibility and availability of population and service providers [4,32]. This method is intuitive while retaining the advantages of a gravity based model [18]. The 2SFCA method has been utilized in a variety of recent studies to measure healthcare services accessibility, e.g., measuring the accessibility of healthcare centers for villagers living in the Indian Alwar district of Rajasthan, the spatial accessibility of primary care physicians and mammography centers for women with breast cancer in the American Appalachian region, and the spatial accessibility of primary healthcare services for Australians [33,34,35]. However, caution is needed while using the method since it makes the unrealistic assumption of equal accessibility for all population living within each catchment area [4].

A distance decay function has been proposed in many studies to model continuous gradual decay within a threshold distance. The distance decay effect is dependent on the phenomena being studied and should be examined separately for each study to account for the different effect distance has on different groups of people for different types of services. Researchers have identified several common forms of the continuous distance decay function: linear decay, Butterworth filter, Gaussian function, inverse-power function, and negative exponential function [36,37]. The enhanced two-step floating catchment area (E2SFCA) method combines a chosen distance decay function with the 2SFCA method [21]. Researchers have used a kernel density function, a Gaussian function, a downward log-logistic function, amongst others, to model the distance decay function [5,38,39,40,41]. However, adjustments still need to be made to the final method to ensure a satisfactory real-world behaviour such as possibly adjusting the radius of a service area or even varying the catchment sizes [40,42,43].

The three-step floating catchment area (3SFCA) method attempts to overcome the possible overestimation of potential population demand in an area with access to multiple service facilities in view of lack of information about potential competition between service facilities by incorporating an extra step to calculate the selection weight in these situations [20]. However, Delamater [38] found that the 3SFCA method produced outcomes that do not necessarily match logic-based outcomes and overestimates the role of competition resulting in both and over- and under- estimation of spatial accessibility for the study area.

Therefore, considering the pros and cons of different approaches as well as the characteristics of our research context, we chose the E2SFCA method as adapted by incorporating a Gaussian distance decay function Equation (4) to estimate geographical accessibility.

The 2SFCA method is essentially a gravity-based accessibility measure of the spatial accessibility to healthcare resources [4,44]. In the first step, all population locations (*k*) that fall within a defined catchment area of a threshold travel time or distance (*d*_0_) around physician location j are used to calculate the physician-to-population ratio (*R_j_*) Equation (1):

(1) $$R_{j}=\frac{S_{j}}{\;\sum_{j\in \left\{d_{kj}\leq d_{0}\right\}}\;P_{k}}$$

In the second step, for each population cluster (*i*), all physician-to-population ratios of facility locations (*j*) within the catchment area of a threshold travel time or distance (*d*_0_) are summed to arrive at the spatial accessibility index of population cluster (*A_i_*) Equation (2):

(2) $$A_{i}=\;\underset{j\in \left\{d_{ij}\leq d_{0}\right\}}{\sum }\;R_{j}=\underset{j\in \left\{d_{ij}\leq d_{0}\right\}}{\sum }\frac{S_{j}}{\sum_{j\in \left\{d_{kj}\leq d_{0}\right\}}P_{k}}$$

Many studies have proposed a distance decay function (i.e., *W_kj_*) to model the continuous gradual decay within a threshold distance, with no effect beyond the threshold distance. A possible model could use a continuous Gaussian distance-decay function. In Equation (3), *β* = *d*_0_/2 and *d_kj_* is the shortest network distance between population location k and physician location *j*:

(3) $$W_{kj}=e^{\frac{-d_{kj}^{2}}{\beta^{2}}}$$

The distance decay function is then added into the 2SFCA method Equation (4):

(4) $$A_{i}=\underset{j\in \left\{d_{ij}\leq d_{0}\right\}}{\sum }\frac{S_{j}}{\sum_{j\in \left\{d_{kj}\leq d_{0}\right\}}P_{k}W_{kj}}$$

Our study adopts an E2SFCA method based on the modified 2SFCA method proposed by Langford, Fry [37] and incorporates a Gaussian distance decay function Equation (4) to calculate geographical accessibility. These parameters will be introduced in the case study below.

## 3. Case Study

### 3.1. Background Information

The Singapore government has implemented several policies in light of city-state’s ageing population, including increased healthcare support through an SGD$8 billion Pioneer Generation Fund [8,45]. All Singapore citizens born on or before the 31st of December 1949 and received their citizenship by 31 December 1986 are eligible for the benefits under the Pioneer Generation Scheme, which covers about 87% of the elderly in Singapore in 2017 [46,47]. With regard to healthcare, pioneers under this scheme can receive an additional 50% off on subsidized services at polyclinics, and special subsidies at private GPs participating in the CHAS.

In Singapore, there are two forms of primary healthcare services: private GP clinics and public polyclinics. Private GP clinics are dispersed in different neighbourhoods across the entire Singapore region. Normally, the patients can see the same doctor continuously, whereas public polyclinics provide subsidized services with less choices and convenience, and the patients are randomly allocated to a duty doctor [48,49,50,51]. It is necessary to note here that the elderly would benefit especially from the continuity of care [52] provided by GP clinics rather than public polyclinics.

Singapore’s Ministry of Health acknowledges that only 20% of primary healthcare services is provided by government polyclinics while the remaining 80% is provided by private practitioners [48]. Although the elderly can already receive 50% off on subsidized services at polyclinics, there are only 18 polyclinics that they can choose from [50,51]. In addition, all Singaporeans and Permanent Residents receive subsidies at polyclinics to a very large extent, making polyclinics an attractive primary healthcare option for most Singaporean residents and increasing competition for medical attention [53]. On the other hand, only pioneers (predefined the elderly) and low-income individuals are eligible for subsidies at Private GP clinics participating in CHAS (henceforth referred to as “CHAS GPs”), with pioneers receiving the most subsidies [54]. Presently, there are 20 polyclinics, 1100 CHAS clinics, and 500 GP clinics [48].

Over the past decade, Singapore has seen an increasing doctor to population ratio, with an all-time high ratio of 1:430 in 2016 [55]. However, in a national survey commissioned by the Ministry of Social and Family Development in 2013, Singaporean elderly said that financial constraint and the lack of clinic or polyclinic near their homes were some of the major reasons for not seeking treatment [56]. While the subsidies under CHAS would ease the financial burden of seeing a doctor, the issue of the proximity to a clinic is still unclear despite the claim that there is a good geographical spread among CHAS GPs and more than 97% of pioneers have “more than one CHAS clinic within 1 km of their homes, or about 15 minutes by public transport” [57]. However, there are some elements that still worry the elderly, such as the difficulty in getting to a bus stop or MRT station, boarding or alighting, and maintaining their balance or obtaining a seat [58,59]. Singapore’s elderly are mobile, with nearly 96% ambulant and physically independent, and 98% ambulant with the help of a walking aid [56]. Thus, if GP services are within walking distance, elderly in Singapore would be more willing to visit a doctor.

Furthermore, the Singapore’s elderly living in HDB flats–Singapore’s public housing–account for 80.6% of Singapore residents aged 65 or above, meaning that this group constitutes the majority of elderly population in Singapore [58]. Given that the average monthly income for HDB flats is less than half of the other dwelling types such as condominiums or landed properties, it stands to reason that elderly individuals living in HDB flats would face more financial constraints while seeking healthcare and would benefit two-fold from the CHAS subsidies, in terms of increased spatial accessibility, and more healthcare service options being made available to them [60].

### 3.2. Data Sources

The datasets used in this case study were provided by various Singapore government agencies—Housing Development Board (HDB), Singapore Land Authority (SLA), and Department of Statistics (SingStat). The unit of analysis used in this study is one HDB block.

#### 3.2.1. Address Points

A shapefile containing all buildings in Singapore linking the address and postal code of the building to its geo-coded location was obtained from Singapore Land Authority (SLA).

#### 3.2.2. HDB Address Points with Flat Type Information

**Figure 1:**
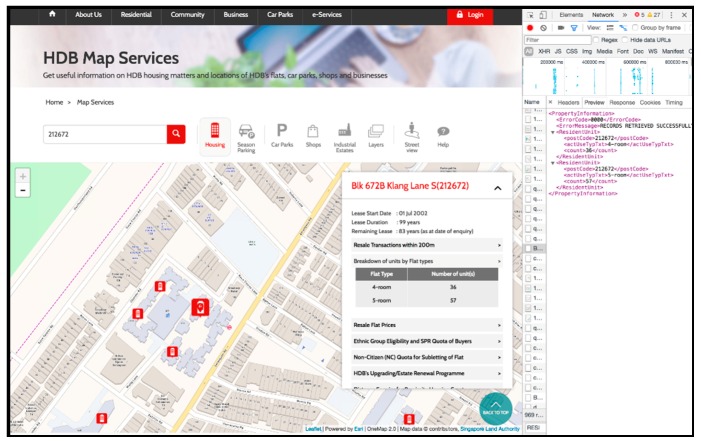
HDB’s map service that shows detailed information about each flat. In the screenshot, a HDB flat with postal code “212672” was selected and the popup showing its information is displayed. The corresponding XML file was located in the Network tab of Developer Tools (Source: https://services2.hdb.gov.sg/web/fi10/emap.html last accessed on 12 November 2017).

HDB flats with flat type information cannot be collected directly nor purchased, but we managed to crawl the data by developing and using a Python script on the HDB website, which provides a map service where users can look at all the HDB flats in Singapore and obtain information about individual flats, such as the postal code and the number of each type of unit (Figure 1). This data is stored in XML files accessible through a URL that can be found in the Network tab under Developer Tools, and is available to anyone who accesses the website.

A Python script was designed to run the URL for all postal codes in Singapore (obtained from the SLA Address Point dataset), read the XML file, and write the information into a csv file. If the building did not exist, then it was excluded from the output csv file. The script had to be run for over 24 h on three computers using PyCharm Community Edition 2017.2.4. Eventually, a csv containing the postal code and the number of each type of unit was produced.

#### 3.2.3. Masterplan 2014 Planning Area

A shapefile containing Singapore’s 2014 masterplan planning areas was obtained from data.gov.sg, a portal launched by the Singapore government to release publicly available datasets from various public agencies.

### 3.3. Data Pre-Processing for Calculating Spatial Accessibility

#### 3.3.1. Location of HDBs in Singapore

**Figure 2:**
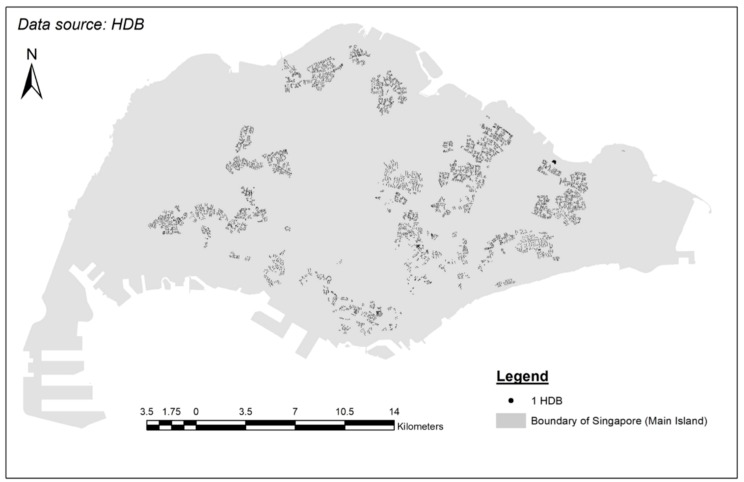
Map of HDBs in Singapore.

A shapefile containing the location of HDBs in Singapore was produced by joining (1) SLA Address Point shapefile, (2) HDB postal codes with flat type information from the HDB website, and (3) URA’s Masterplan 2014 Planning Areas. The map of all the HDBs can be seen from Figure 2.

#### 3.3.2. Population Density of Singapore Elderly Living in HDBs

**Figure 3:**
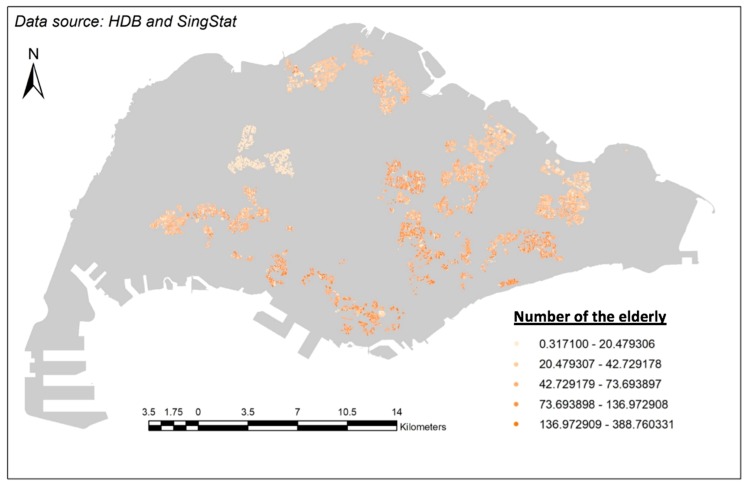
Density map of elderly living in HDB flats in Singapore.

A shapefile containing the population density of Singaporeans aged 65 and over per HDB block was produced using (1) a SingStat dataset categorizing the Singapore resident population by planning area, age group and type of dwelling in June 2017 and (2) URA’s Masterplan 2014 Planning Areas. The map of the distribution of the elderly living in HDB flats can be seen from Figure 3.

#### 3.3.3. Parameter Setting

In this study, A_i_ is the spatial accessibility index for a HDB at location *i*. *P_k_* is the number of the elderly in a HDB flat at location *k*. *S_j_* is assumed to be a constant value of 1 for all CHAS GPs because it is the minimum number of doctors that can possibly be available at each CHAS GP, ensuring there will not be an over-calculation of the physician-to-population ratio. *d_kj_* is the distance between a CHAS GP at location *j* and a HDB flat at location *k*.

The threshold distance (*d*_0_) was set at 400 m because the elderly prefer to live within 1 km of a healthcare center, and the distance covered by healthy Singaporeans up to age 85 in an assessment of their functional exercise capacity was 560–105 m, independent of age. The lower end of this range is 455 m. However, since no network dataset was used and it can be assumed that the route the elderly take to reach their chosen CHAS GP will not always be a straight line, 455 m was rounded down to 400 m. A distance threshold rather than a travel-time threshold was chosen because the elderly walk at a varied pace but, if healthy and willing, they are able to cover a certain distance in a given time [61,62,63,64,65].

### 3.4. Results

**Figure 4:**
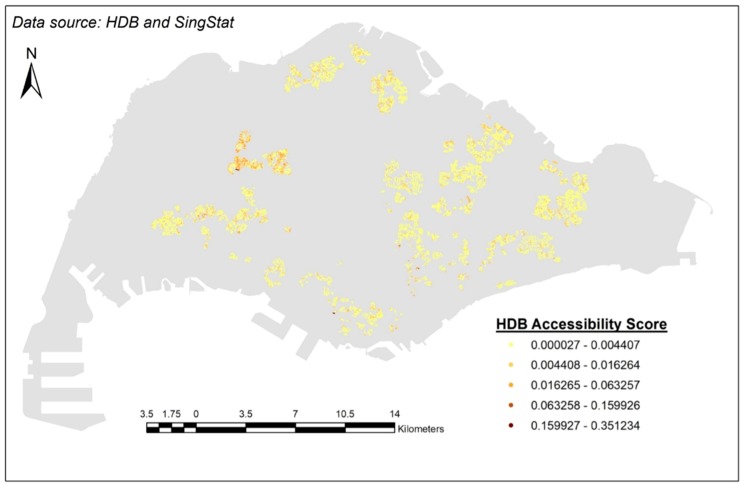
The *A_i_* values of each HDB flat.

The *A_i_* values ranged between 0.000027 and 0.351234. Figure 4 is a thematic map showing the *A_i_* values; a darker shade of color represents a higher *A_i_* value, and a lighter shade of color represents a lower *A_i_* value.

A hot spot analysis was then performed on the A_i_ values. The Getis-Ord Gi* statistic for each HDB flat was calculated to find statistically significant hot spots of high or low value spatial clusters based on z-scores and *p*-values.

**Figure 5:**
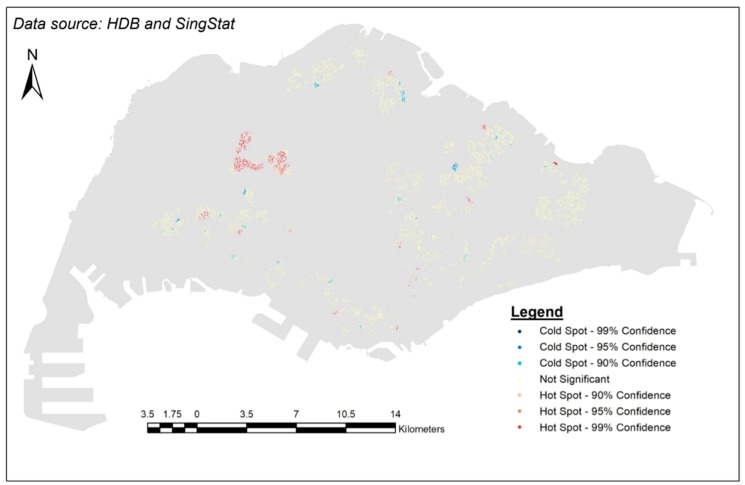
Results of the hot spot analysis.

The result from hot spot analysis is shown in Figure 5, where the blue areas represent statistically significant clusters of cold spots, and the orange/red areas represent statistically significant clusters of hot spots. Within the clusters of cold spots, the lightest blue indicates a confidence level of 90%, while the darkest blue a confidence level of 99%, and so on. The yellow points represent areas where no statistically significant clusters are found.

## 4. Discussion

**Figure 6:**
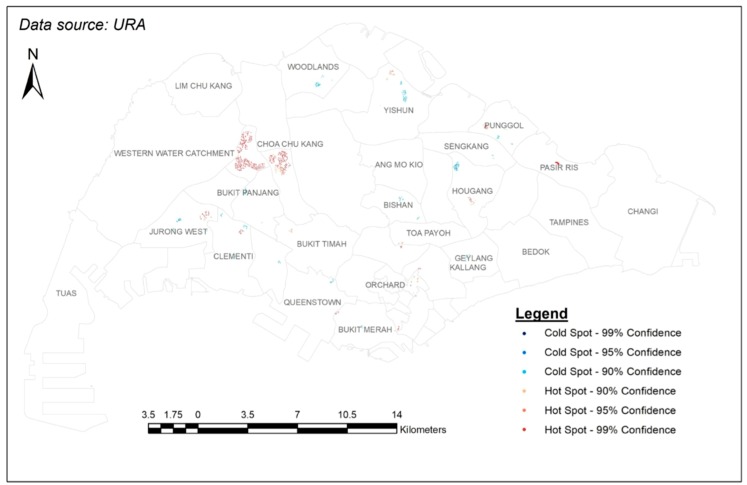
Results of hot spot analysis against the planning boundaries of Singapore.

For further analysis and discussion, we produced a map demarcating the planning boundaries and displaying only the clusters of hot and cold spots (Figure 6).

### 4.1. Explanations on the Hot Spots

The hot spots could be a result of the maturity of the HDB Town. These spots are usually located in or near to towns and estates that were developed before the 1980s, In fact these towns are regarded as Mature Towns/Estates [66]. The Mature category includes areas such as Queenstown, Toa Payoh, Clementi, Kallang, and the Middle-Aged category includes areas such as Jurong West, Bukit Panjang, Choa Chu Kang, Hougang and Bukit Timah. In the map, these areas do have a cluster of hot spots, possibly due to the organic growth of businesses in the area over the years. Understandably it also includes GPs, and has a higher possibility of GPs in the area joining CHAS due to the larger number (competition per se) of GPs. Interestingly, some of these hot spots are very close to where the first HDB towns were built in the area. For example, the hot spot between Queenstown and Bukit Merah is located near Forfar Heights, which stands in the place of Forfar House, the very first high-rise apartment block built in 1956 [67,68]. Another example would be the hot spot in the north of Yishun, which is located very close to the Chong Pang area, where the first HDB flats were built in the 1980s [67].

Another reason for the hot spots could be the government’s setting up of rejuvenation projects in the area. The Singapore government announced the Remaking Our Heartland (ROH) initiative in 2007 to help mature HDB towns keep up with modern designs [46]. This initiative tailors renewal plans based on the needs of the community and ensures that the areas remain relevant and sustainable while still maintaining their unique characteristics [69]. Areas like Punggol and Yishun were part of the first batch of the chosen towns in 2007, and Hougang was part of the second batch in 2011. An outcome of this initiative is that more GPs moved to these areas attracted by new facilities such as community plazas and the enhancement of community and connectivity. This would cause an increase in the number of people who would potentially visit the community plaza, thereby providing the GPs with a larger potential consumer market [70]. Such an increased number of GPs would once again result in more GPs in the area joining CHAS. With the ongoing and future projects under the ROH initiative, this could attract more GPs to new areas and provide more primary healthcare services.

### 4.2. Explanations on the Cold Spots

**Figure 7:**
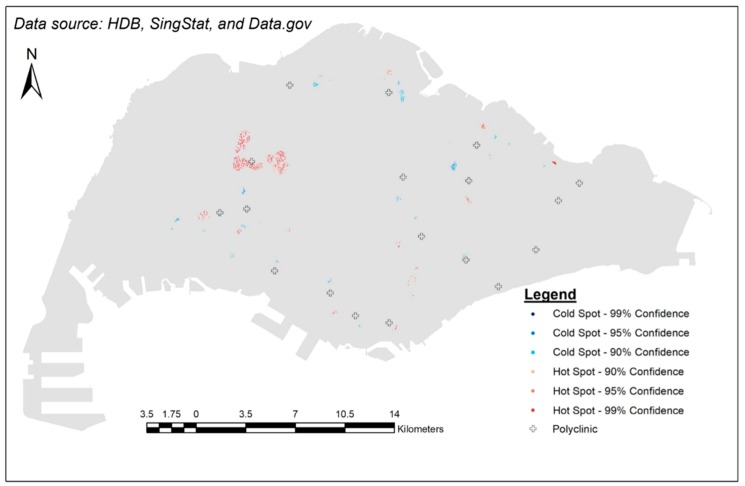
Results of hot spot analysis against locations of polyclinics.

As for the cold spots, one very compelling reason could be the locations of polyclinics. The spread of polyclinics does seem to match the spread of cold spots (Figure 7). As mentioned earlier, it would be cheaper for elderly individuals to visit a polyclinic (further 50% subsidy on the already subsidized services). Thus, it makes sense that GPs would try to avoid being close to them. On the other hand, GPs may not have sufficient number of patients to join CHAS. This would then result in fewer GPs in the area participating in CHAS (cold spots). It is quite likely that additional incentives are needed if the government wants to encourage more GPs in the area to participate in CHAS.

Another reason for cold spots could relate to the government’s city planning. For example, Punggol is considered a “young” area because it was one of the few areas that had build-to-order (BTO) HDB flats in around 2011. Since eligibility to purchase requires at least a nuclear family, the demographics of the area tend to be young families and their children. This is actually echoed in the figure where more than 11% of Punggol’s residents are aged 4 and below [47,71]. Consequently, the infrastructure of the neighbourhood is featured with 52 childcare centers and two mega childcare centers with 1000 places in each [71]. This concentration of efforts for a certain demographic group may also mean overlooking the other, namely the elderly. A more balanced and comprehensive town planning may be required while planning future new towns.

### 4.3. More Implications

In this study, GIS concept and methods have been utilized to measure geographical accessibility of one major type of primary healthcare services, i.e., CHAS GPs, to a scale of HDB flats, i.e., the elderly living in HDB flats. This has worked better than in most other studies using the centroid of a census population tract to indicate the demand for all individuals within the same catchment area [4,18,43,44,72]. This advantage is a direct outcome of the finer scale this research has reached. Therefore, the results are considered more reliable for policy makers in the relevant fields. In the short term, more efforts could be put into convincing and incentivizing GPs located near areas with clusters of low accessibility scores to participate in CHAS so as to improve the elderly’s geographical accessibility to a GP at a subsidized cost. In addition, it should be noted that even within the areas that do not have statistically significant clusters of high or low values, there are still individual places that may have low accessibility scores. These findings can also contribute the decision making process in terms of directing the current and future efforts in CHAS.

On the other hand, geographically (according to the map) more specific efforts should be put into increasing the number of CHAS GPs in certain areas. It is worth noting that cold-spot areas such as Woodlands, Hougang/Sengkang/Punggol, Geylang, and Bishan/Ang Mo Kio are quite far away from any hot spots, making it quite impossible to draw on the resources in the hot-spot areas. It is important to note that in a small city-state like Singapore, although planning boundaries were used to direct attention to specific hot or cold spots, people may not view area boundary as the administratively determined boundary. This could contribute to the existence of both hot and cold spots within one planning boundary. There is a need for greater flexibility with the administrative arrangement so that elderly citizens may draw on a nearby yet different administrative area.

In the long term, based on the hot spot analysis or accessibility scores, follow-up research efforts should be put into the optimization study to find out the best locations to set up GPs who are considering setting up new service centers. Carrying out a hot spot analysis on a regular basis, such as once per year, has the potential to inform/update the status of gaps between the demand from the elderly and the supply from existing CHAS GPs.

### 4.4. Limitations

One major limitation of the study relates to the distance decay function used, which reflects the willingness to access a medical service and its determinant should be a function of a variety of factors that is unique to the population under study. Ideally, the distance decay function should be generated following an empirical investigation possibly applying regression methods on the data collected. However, such a dataset was not available for this study, so a mathematical model had to be used in this study as in many other studies [18,33,73,74,75,76,77,78]. Secondly, this study is cross-sectional rather than a longitudinal study. There certainly are some dynamic factors that may bring about very different results over time. Among them are targeted healthcare policy, increased health hazards caused by global warming, and change in economy that may affect the contribution of income and savings to elderly parents. These are all beyond the scope and control of this study.

## 5. Conclusions

Rapid ageing is a major regional if not global demographic challenge; it is not just faced by Singapore, but many other countries. Primary healthcare services are critical to the elderly, and it is important not only to have the concerned services available, but also has to be geographically accessible. This study has examined the geographical accessibility of one representative healthcare service, i.e., CHAS GPs, for the elderly living in HDBs at the block level in Singapore by employing an enhanced 2-step floating catchment area method and a Gaussian distance-decay function in combination with the GIS technology. This research has succeeded in reflecting the current status of the geographical accessibility of CHAS GPs for elderly individuals living in HDBs across the entire Singapore. Areas with relatively weak geographical accessibility of CHAS GPs have been spotted, explained and discussed. Suggestions have been put forward for policy makers, and the value of using innovative technology and approaches has been demonstrated, along with recommendation for future studies. In the long run, the spatial analysis should also be repeated on a regular basis for better understanding the change in geographical accessibility of CHAS GPs alongside the change of the elderly demographics in Singapore. Similar geographical accessibility studies could be conducted with respect to a variety of healthcare resources such as general hospitals, polyclinics, post-acute care, with support from GIS, for meeting the needs of the elderly and other specific groups.
